# Enhancing nutritional value and health benefits of gluten-free confectionery products: innovative pastilles and marshmallows

**DOI:** 10.3389/fnut.2023.1321004

**Published:** 2024-01-12

**Authors:** Yuliya Pronina, Olga Belozertseva, Zhanar Nabiyeva, Annachiara Pirozzi, Serena Carpentieri, Giovanna Ferrari, Elmira Bazylkhanova, Anastasiya Burlyayeva

**Affiliations:** ^1^Department of Food Technology, Almaty Technological University, Almaty, Kazakhstan; ^2^Department of Industrial Engineering, University of Salerno, Fisciano, Italy; ^3^ProdAl Scarl, University of Salerno, Fisciano, Italy

**Keywords:** confectionery products, pastille, marshmallow, bioactive compounds, probiotic

## Abstract

**Introduction:**

The research focuses on enhancing the nutritional value and potential health benefits of gluten-free confectionery products, developing innovative pastilles and marshmallows enriched with medicinal herb extracts, probiotics, and bioactive compounds from natural sources.

**Methods:**

Physicochemical properties, including water activity, texture, and color, are assessed to evaluate the quality of the final products. Moreover, *in vitro* digestibility of the confectionery products is also investigated, with a focus on the release of bioactive compounds such as total phenolic compounds (TPC) and total anthocyanin (TAC) during simulated gastrointestinal digestion.

**Results and discussion:**

Results indicate that the addition of specific ingredients to pastille samples does not lead to variations in water activity (~0.44), preserving the original properties, quality, and stability of the food. In contrast, the incorporation of additives in marshmallow products significantly increases water activity (*p ≤ 0.05*), attributed to their moisture-retaining effect. In general, our findings reveal that texture properties and color parameters are significantly affected by different formulations (*p ≤ 0.05*) for both confectionery products. Notably, the use of fruit and berries puree, along with the incorporation of additives, improves the functionality of confectionary products in terms of consumer acceptance (harder pastilles and softer marshmallow) and product quality. Furthermore, the study reveals that bioactive compounds are released and become more bioaccessible during digestion, particularly in the intestinal phase, with a maximum release exceeding 97% of TPC and TAC for both pastille and marshmallow samples. These findings pave the way for the development of a new category of gluten-free confectionery products, enriched with functional ingredients that offer potential health benefits, aligning with consumer preferences for natural, functional, and health-conscious treats. This research contributes to the evolving the landscape of functional confectionery products and underscores their potential as immune-boosting and naturally based food options.

## 1 Introduction

Confectionery products, usually referred to a large range of food items, such as candy, caramel, toffees, marshmallow, fondant, jelly, tablets, or chewing gum (1), are favored foodstuff of consumers of all age groups, especially children, given their interesting colors and shapes, as well as unique flavor and textural properties (2). Confectionery primarily consists of sugar, water, various hydrocolloid-based gelling agents, stabilizers, fats, emulsifiers, colorants, flavors, acids, as well as nuts and/or fruit-based flavorings/seasonings (juices, concentrates, purees, jams) used especially as filling materials (3). Among these components, hydrocolloids such as gelatin, starch, and pectin act as the basic gelling and stabilization agents, ensuring the formation of a stable network structure and determining the characteristics of the final product (4). Complementary ingredients, such as antioxidants, humectants, and organic acids, enhance product stability (5); while sensory attributes are attended to through the incorporation of edible oils (6), diverse fillings (7), colorants (8), and flavors (9). Since the pivotal ingredient employed in confectionery formulations is the sugar (sucrose), the categorization of confectionery can be delineated according to the state of sugar present within it (9), including (i) non-crystalline (liquid, amorphous, and glassy); (ii) partially crystalline; and (iii) crystalline.

Within the realm of confectionery, pastilles and marshmallows are classified as amorphous products based on their sweetener composition. Pastilles are obtained by blending finely powdered sugar with natural fruit and herbal extracts (10). Marshmallows, instead, are aerated confectioneries with a foam-like structure, stabilized by the addition of proteins, such as gelatin, gum arabic, or egg albumen (11, 12) often combined with fruit and berry puree, usually strawberries, raspberries, or citrus fruits. Therefore, two important components in marshmallows’ structure are air bubbles, which increase volume and improve texture, and moisture, which controls the viscosity of the product and facilitates the air incorporation to the mass during the aeration process (13).

Despite their delightful appeal, confectionery products, including pastilles and marshmallows, contain a significant amount of fats and carbohydrates, making them highly caloric foods. This association with calorie intake raises concerns about obesity (14, 15), which is a highly diffused disease at present days. Thus, there is a pressing need to develop sugar-free and low-calorie confectionery (16), preserving the quality characteristics, especially in terms of sensory (color), taste and aroma (flavor), and textural aspects which are critical factors for consumer acceptance and success of these new products (17). Furthermore, the confectionery industry’s supply of its products to the global market requires effective stability of quality characteristics throughout the shelf life (18). In light of these considerations, the incorporation of functional ingredients, such as vitamins, minerals, and even anthocyanins, polyphenols, and tocopherols, with beneficial effects on human health, can be seen as an opportunity for the confectionery industry (19). In particular, the consumer’s demand is shifting toward functionalized confectionery products containing natural compounds, free from synthetic molecules (18), low in calories and enriched with active ingredients with health benefits and colored with natural pigments were also proposed (20, 21). Indeed, food additives are perceived as compounds of health concerns. For instance, hyperactivity, kidney damage are often associated to many azo dyes (9). Notably, the use of plant medicinal raw materials or extracts represent a promising and suitable pathway for enhancing the nutritional and health attributes of confectionery products (22). Rich in vital components such as vitamins, minerals, antioxidants, and phenolic compounds, these materials offer preventive effects against various viral and non-infectious diseases (23), making them very valuable for functional nutrition bolstering immunity. Different studies highlighted the potential of medicinal plants, containing flavonoids, alkaloids, tannins among others, in the treatment of viral diseases like SARS-CoV-2 and MERS-CoV (24). Therefore, recent research efforts focused on identifying local medicinal herbs for enriching protein confectionery products, with a primary criterion being their content of vitamins, polyphenols, and antioxidants. For example, *Hypericum perforatum* L. (common name St. John’s wort) is a medicinal herb found mostly in the Mediterranean region and used in different studies as a curative plant against various diseases such as antimicrobial (25, 26), anticancer (27, 28), antioxidant effects (29), and obesity (30). Moreover, Pronina et al. (31) were proven that hypericum herb and *Hippophae rhamnoides* L. (common name sea buckthorn leaves) are rich sources of vitamins B2, B3, B5, B6, C, as well as polyphenols and antioxidants (31). Based on these findings, these herbs were used as ingredient to produce an immunity-supporting marmalade (32). The resulting product showed excellent organoleptic properties and a high concentration of water-soluble vitamins, polyphenols, and antioxidants (32). Apart from that, *Rosa canina* L. (common name rosehip fruit) is another high functional medicinal plant material with positively effects on health thanks to the high content and variety of phytochemicals. Several studies have reported its preventive and curative effects for memory disfunction (10.1113/EP087535), cancer (33, 34), and anti-inflammatory, antinociceptive and antioxidant activities (35, 36). In addition to the aforementioned medicinal herbs, probiotic microorganisms are living bacteria and yeasts which can be incorporated in food products as complementary therapies (37) to (i) enhance immunity, (ii) reduce inflammation, (iii) improve tolerance to stress and unfavorable environmental conditions, (iv) relieve gastrointestinal pain, and (v) prevent diarrhea.

To fully grasp the implications of this enrichment, the current study delves into the preservation of physicochemical properties and digestibility of innovative and functionalized confectionary products. Within this framework, alternative gluten-free pastilles and marshmallows based on fruit and berries enriched with medicinal herb (sea buckthorn leaves, hypericum herb, and rosehip) and probiotics have been produced with the attempt of improving the quality and nutritional profile of these food products.

## 2 Materials and methods

### 2.1 Raw materials and chemicals

*Golden Superior* and *American apples* varieties and low-esterified pectin were kindly provided by the FruktProduct company (Almaty, Kazakhstan) and NovaProduct company (Almaty, Kazakhstan), respectively. Berries fruits and albumin were purchased from a local company (Almaty, Kazakhstan). Medicinal herbs, i.e., St John’s Wort, sea buckthorn leaves, and rosehip were purchased from a local pharmacy (Almaty, Kazakhstan), whose vitamin profile and bioactivity have been reported in Pronina et al. (31). The probiotic (Maxilin^®^), a mixture of egg yolk, egg white and *Lactobacillus acidophilus* strain bred from meconium, was kindly provided by EnergyMax Group (Semey City, Kazakhstan).

Citric acid (E330), α-amylase (A3176, ≥ 5 U/ mg) from porcine pancreas, pepsin (P7000, 674 U/mg) from porcine gastric mucosa, pancreatin (P3292, 4 × USP) from porcine pancreas, bile salts (B8756), and all the chemicals and reagents used for the analyses were purchased from Sigma Aldrich (Steinheim, Germany).

### 2.2 Pastille samples preparation

**Figure 1:**
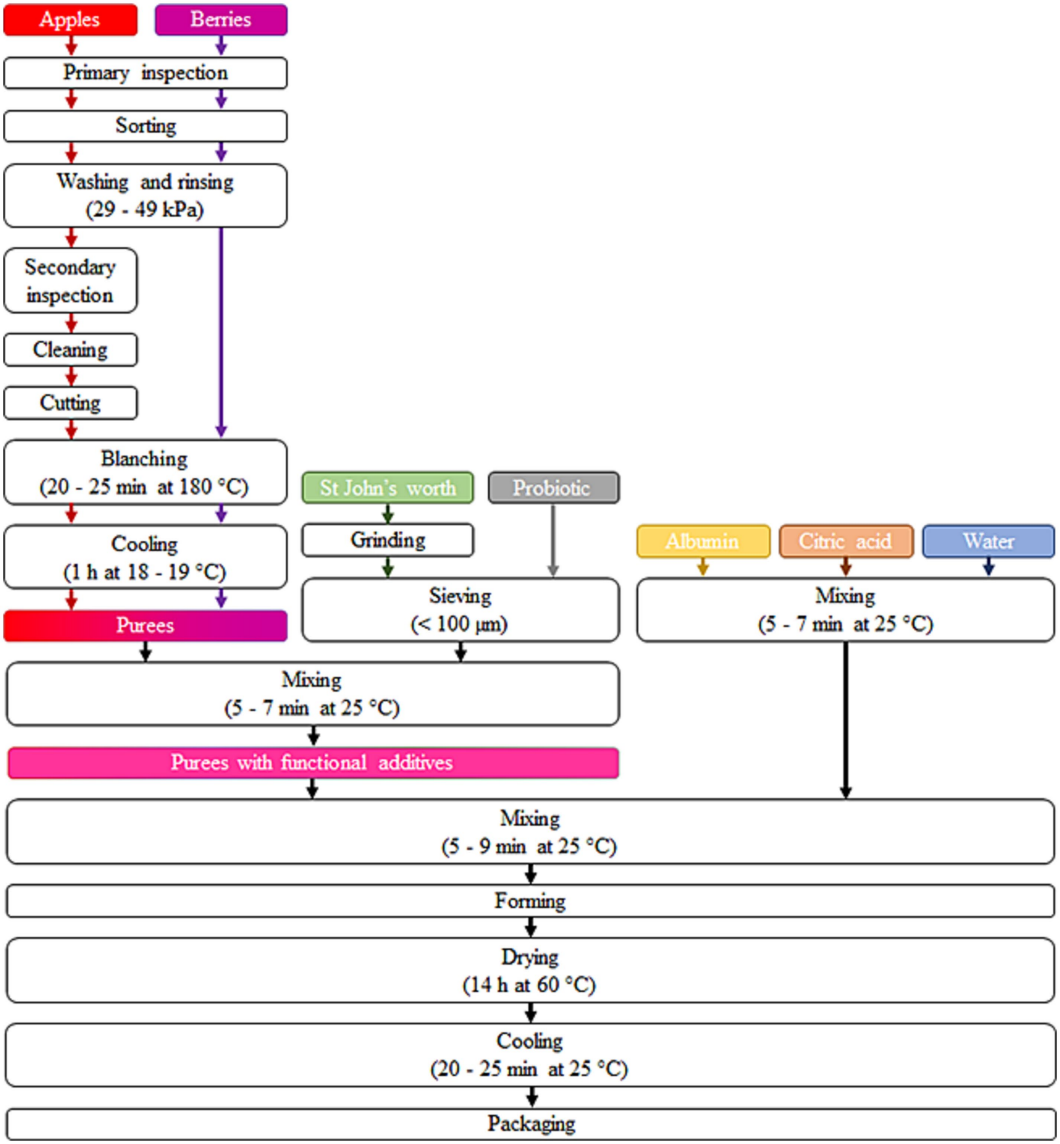
Production steps of fruit and berries based functionalized pastilles.

Three types of pastille samples were produced using the methodology outlined in Figure 1 and with compositions (% on dry matter, DM) detailed in Table 1: (i) *P_AC* prepared from apple and cranberry puree; (ii) *P_AC + S* prepared from apple and cranberry puree with the addition of sea buckthorn leaves and St John’s worth; and (iii) *P_AC + SP* prepared from apple and cranberry puree with the addition of sea buckthorn leaves and St John’s worth and the probiotic.

**Table 1:** Composition of functionalized pastille and marshmallow samples (%).

	P_AC	P_AC + S	P_AC + SP	M_A	M_S + R	M_AC + S	M_AB + S
Apple puree	68.50	68.02	67.67	32.40	–	16.11	16.00
Cranberry puree	7.61	7.56	7.52	–	–	16.11	–
Strawberry puree	–	–	–	–	32.00	–	–
Blueberry puree	–	–	–	–	–	–	16.00
Water	21.31	21.16	21.05	12.97	12.85	12.87	12.83
Albumin	2.43	2.41	2.40	1.30	1.20	1.28	1.28
Sugar	–	–	–	51.85	51.35	51.48	51.24
Agar	–	–	–	1.30	1.20	1.28	1.28
Citric acid	0.15	0.15	0.15	0.098	0.098	0.098	0.098
Salt	–	–	–	0.082	0.082	0.082	0.082
St John’s worth	–	0.35	0.35	–	–	0.69	–
Sea buckthorn leaves	–	0.35	0.35	–	–	–	1.19
Rosehip	–	–	–	–	1.22	–	–
Probiotic	–	–	0.51	–	–	–	–

P_AC, P_AC + S, and P_AC + SP refer to pastille samples prepared with apple and cranberry puree, apple and cranberry puree plus St John’s worth and sea buckthorn leaves, apple and cranberry puree plus St John’s worth, sea buckthorn leaves, and probiotic, respectively. M_A, M_S + R, M_AC + S, and M_AB + S refer to marshmallow samples prepared with apple puree, strawberry puree plus rosehip, apple and cranberry puree plus St John’s worth, and apple and blueberry puree plus sea buckthorn leaves, respectively.

The optimal probiotic concentration of 0.021% with a lactic acid microorganism live cell count of 10^8^–10^10^ CFU/g and probiotic viability of 63–88% (38), was selected based on preliminary tests demonstrating that the probiotic percentage increased, the porosity of the pastille samples was adversely affected, resulting in a significant deterioration of the rheological properties of the product.

The produced pastille samples were stored under refrigerated conditions (T = 4°C) until further analyses. The dry matter of apple, strawberry, cranberry, and blueberry purees was 13.7 ± 2%, 14.1 ± 2%, 10.5 ± 2%, and 12.7 ± 2%, respectively.

### 2.3 Marshmallow samples preparation

**Figure 2:**
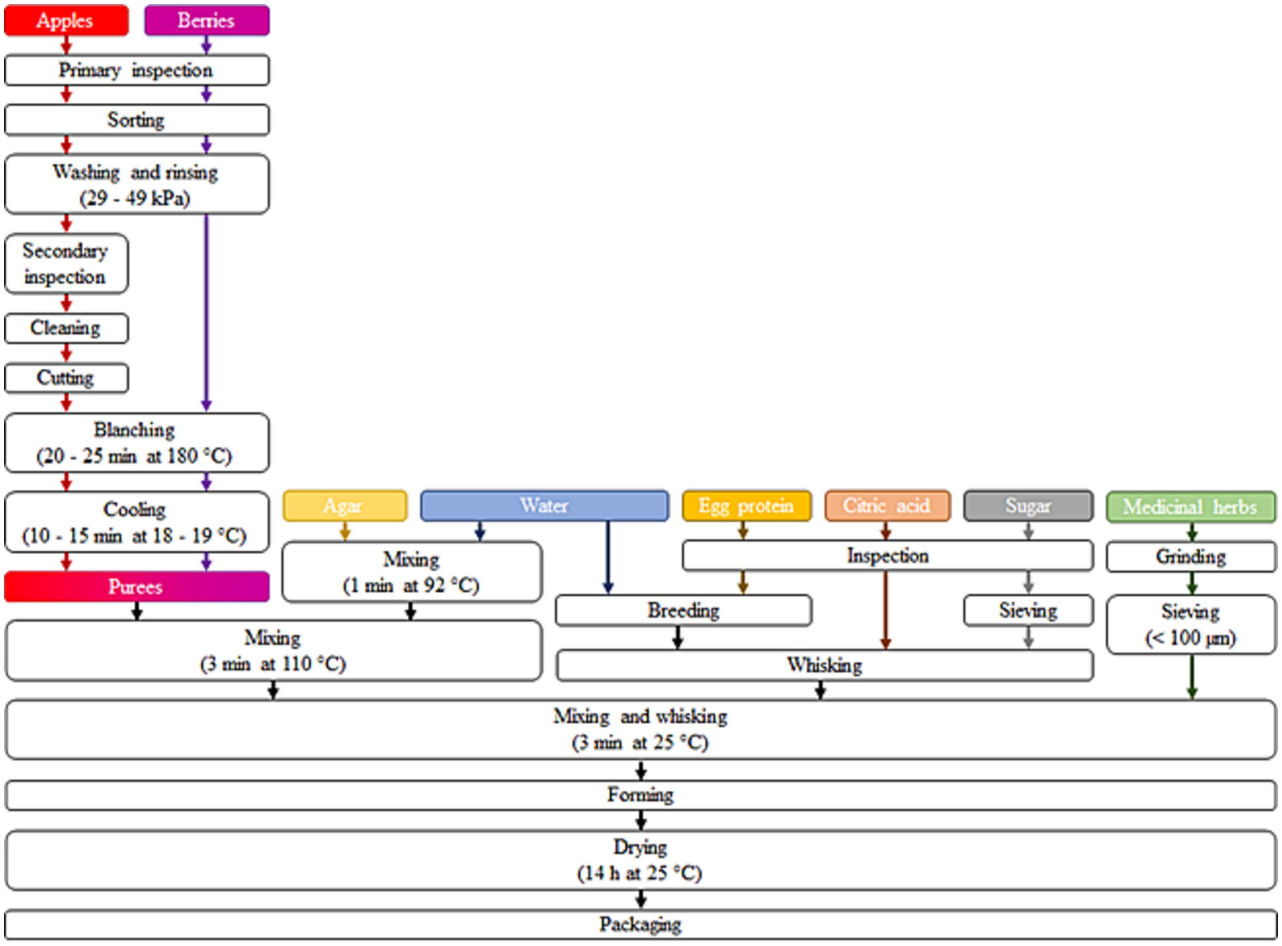
Production steps of fruit and berries based functionalized marshmallows.

Four types of marshmallow samples were produced using the methodology outlined in Figure 2 and with composition (%_DM_) detailed in Table 1: (i) *M_A* prepared from apple puree; (ii) *M_S + R* prepared from strawberry puree with the addition of rosehip; (iii) *M_AC + S* prepared from apple and cranberry puree with the addition of St John’s worth; and (iv) *M_AB + S* prepared from apple and blueberry puree with the addition of sea buckthorn leaves.

The produced marshmallow samples were stored under refrigerated conditions (T = 4°C) until further analyses. The dry matter of purees used for marshmallows preparation was measure in triplicate on independently prepared samples using a digital refractometer (Atago PAL-α digital refractometer, Milan, Italy), and the result reported as means ± standard deviations (as detailed in paragraph 2.2).

### 2.4 Proximate composition of medicinal herbs and pastille and marshmallow samples

Moisture and ash content was determined through the gravimetric method at 105°C (AOAC 950.46) and 525°C (AOAC 923.03), respectively, until a constant weight. Protein and fat contents were determined according to AOAC 920.152 (micro-Kjeldahl method, conversion factor 6.25) and AOAC 920.39, respectively. Total carbohydrates were evaluated by subtracting the sum of the amount of the other proximate components to 100 g of sample.

### 2.5 Physicochemical characterization of pastille and marshmallow samples

#### 2.5.1 Water activity analysis

The water activity (a_w_) of the samples was measured in triplicate using the a_w_ meter (AQUALAB 4TE, MeterFood, United States) according to the method used by (39).

#### 2.5.2 Texture analysis

The texture profile of the samples was analyzed according to the method described by Bourne (40), by using a Texture analyzer (Stable Micro Systems, United Kingdom) equipped with a stainless-steel needle probe. From the resulting force-time curve, texture parameters, namely hardness (N; maximum peak force during the first compression cycle), adhesiveness (N·s; negative force area for the first bite), and cohesiveness (−; ratio of positive force area during the second compression to that during the first compression) were evaluated.

#### 2.5.3 Color analysis

Color parameters of samples (L*: brightness, a*: ± red-green and b*: ± yellow-blue) were determined using a colorimeter (Chroma Meter CR-400, Konica Minolta, Japan), as described by Pirozzi et al. (41). Chroma (C*), hue angle (h°), and color variation (ΔE) were calculated using the Equations 1-3:


(1) $$C^{*}=\sqrt{{a^{*}}^{2}+{b^{*}}^{2}}$$

(2) $$h{}^{\circ}=\arctan\frac{b^{*}}{a^{*}},\mathrm{if}\;a^{*}\mathrm{and}\;b^{*}\mathrm{are}\;\mathrm{both}\;\mathrm{positive}$$

(3) $$\Delta E=\sqrt{{\left(\Delta L^{*}\right)}^{2}+{\left(\Delta a^{*}\right)}^{2}+{\left(\Delta a^{*}\right)}^{2}}$$

### 2.6 *In vitro* gastrointestinal digestion

The simulated gastrointestinal digestion was assessed on the investigated samples following the procedure described by Carpentieri et al. (42), with slight modifications.

#### 2.6.1 Oral phase

A complete simulation of the mastication and swallowing processes was implemented. Briefly, 30 g of marshmallow and pastille samples were mixed with a volume of simulated salivary fluid (SSF; S/L ratio of 1:2 for marshmallow samples, and S/L ratio of 1:6 for pastille samples), consisting of 5 g/L of α-amylase, 0.117 g/L of sodium chloride, 0.149 g/L of potassium chloride, 2.1 g/L of monosodium carbonate, and 0.074 g/L of calcium carbonate. Prior to the oral phase digestion step, the samples were cut in 1 cm cubes. The samples were then subjected to a physical disruption process in a controlled homogenizer (Stomacher 400, Steward, England) at 200 rpm for 1 min.

#### 2.6.2 Gastric phase

The sample from the oral digestion step was mixed (1:1 ratio) to the simulated gastric fluid (SGF), consisting of pepsin solution (3.2 g/L), 2 g/L of sodium chloride, and 7% *v/v* hydrochloric acid 37%. Then, the mixture was adjusted to pH 3 with 1 M hydrochloric acid and incubated at 37°C for 120 min under continuous stirring at 130 rpm (Orbital incubator SI50 system, Bibby Sterilin LTD, United Kingdom).

#### 2.6.3 Intestinal phase

The sample from the gastric digestion step was mixed (1:1 ratio) to the simulated intestinal fluid (SIF) consisting of 5 g/L of pancreatin, 0.2 g/L of calcium carbonate, 0.2 g/L of calcium chloride, 1.75 g/L of sodium chloride, and 25 g/L of bile salts. The pH was adjusted to a final value of 7 by the addition of hydrochloric acid (0.1 M) and sodium hydroxide (0.05 M), and incubated at 37°C for 120 min under continuous stirring at 130 rpm.

### 2.7 Analytical determinations

#### 2.7.1 Total phenolic content

Total phenolic content (TPC) of the samples was determined using the Folin-Ciocalteau method as reported by Carpentieri et al. (43). Results were expressed as milligrams of gallic acid equivalents (mg_GAE_) per g_DM_ of the raw material.

#### 2.7.2 Ferric reducing antioxidant power

FRAP assay of the samples was carried out according to the method described by Pirozzi et al. (17, 44). Results were expressed as milligrams of ascorbic acid equivalents (mg_AAE_) per g_DM_ of the raw material.

#### 2.7.3 Total anthocyanin content

Total anthocyanin content (TAC) of the samples was determined using the pH differential method described by Carpentieri et al. (45) with slight modifications. Briefly, two mixtures were prepared per each extract by diluting, with a dilution factor equal to 2, one sample with pH 1.0 buffer (0.19% *w/v* of potassium chloride in water) and the other with pH 4.5 buffer (5.44% *w/v* of sodium acetate in water). The absorbance of the diluted reacting solutions was then measured at 520 and 700 nm using a V-650 spectrophotometer (Jasco Inc. Easton, MD, United States) within 30 min of their preparation. Results were expressed as milligrams of cyanidin-3-glucoside per g_DM_ of the raw material.

### 2.8 Release of bioactive compounds during i*n vitro* digestion

Samples, which were withdrawn after the oral phase, 60 and 120 min of gastric phase, 40, 80, and 120 min of intestinal phase, were centrifuged at 25000 × g at 4°C for 20 min using an Eppendorf centrifuge (Micro Centrifuge 5417R, Eppendorf Srl, Milan, Italy). TPC were determined as described in 2.6.1. TPC release from the samples was determined according to the Equation 4:


(4) $$\mathrm{Releas}e_{TPC}=\frac{TPC_{i}}{TPC_{T}}\cdot 100$$

where TPC_i_ is the phenolic content detected in each sample during the digestive phases, and TPC_T_ is the total phenolic content measured in the sample taken after 150 min of the intestinal phase, assuming that, 30 min after the intestinal phase, the complete disintegration of the matrix occurred.

The same procedure was used to evaluate the release in terms of antioxidant activity and TAC from the investigated samples.

### 2.9 Sensory analysis

Sensory evaluation was carried out by a panel of 15 subjects. The samples were scored using a standard five-point scale from 1 (the lowest grade) to 5 (the highest grade) according to GOST6441-2014. The individual quality features were evaluated using the following factors: taste and odor (type and desirability), color (uniformity), consistency (softness, stiffness), structure/shape (uniformity), surface (uniformity), and overall impression. The final scores are given as the arithmetic mean of the different factors.

### 2.10 Statistical analysis

All the experiments and analyses were carried out in triplicate and the results were shown as means ± standard deviations. Differences (*p ≤ 0.05*) between mean values were analyzed with parametric test one-way ANOVA with Tukey’s *post-hoc* test using SPSS 20 (SPSS IBM, Chicago, United States) statistical package.

## 3 Results and discussion

### 3.1 Physicochemical properties

**Figure 3:**
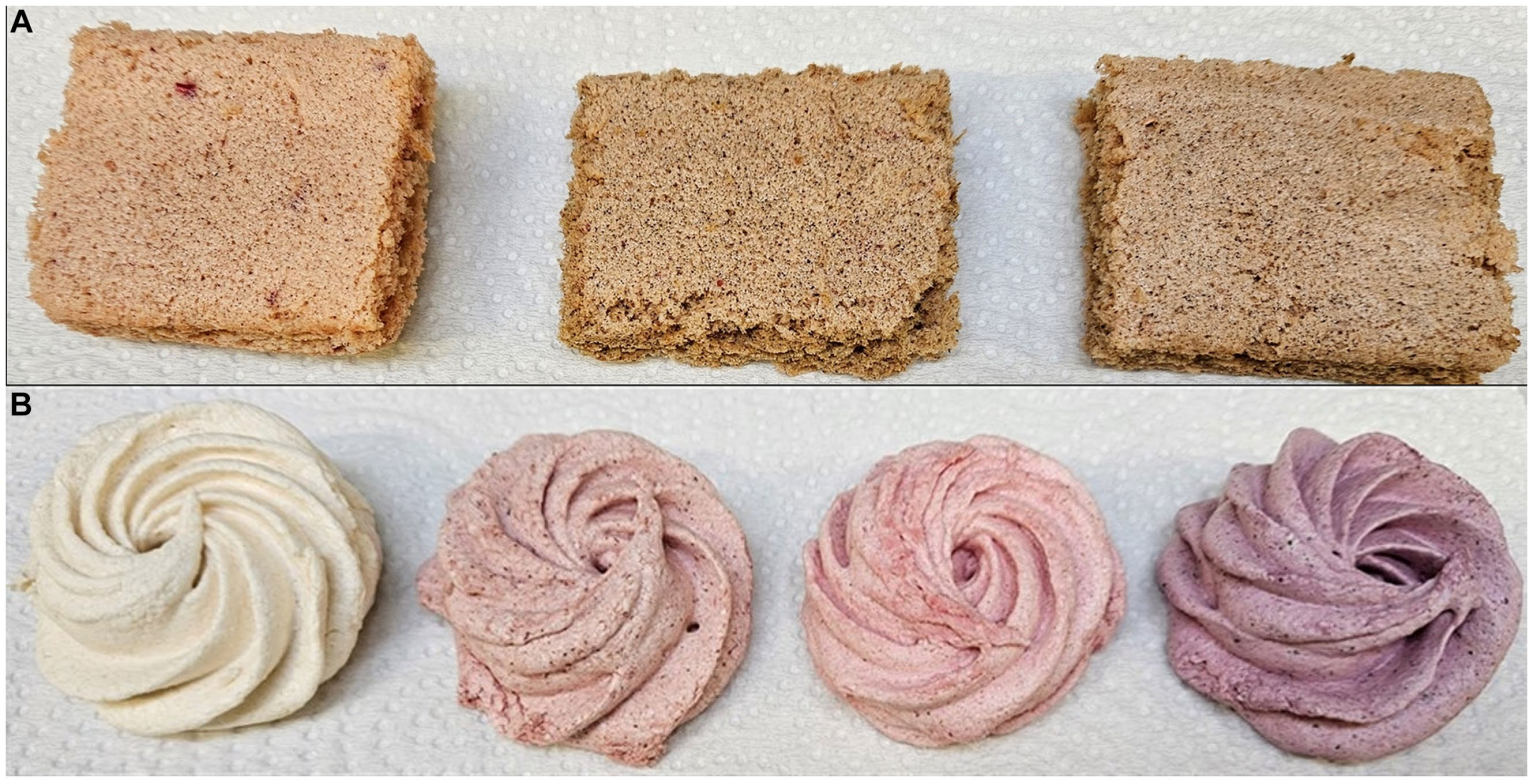
Picture of functionalized confectionary food products: **(A)** pastilles and **(B)** marshmallows.

The characteristics of medicinal herbs and pastille (Figure 3A) and marshmallow (Figure 3B) samples are presented in Table 2.

**Table 2:** Proximate composition of medicinal herbs and pastille and marshmallow samples.

Sample	Moisture (%)	Ash content (%)	Proteins (g)	Fats (g)	Carbohydrates (g)	Energy	
						(kcal)	(kJ)
St John’s worth	6.43 ± 0.09^a^	3.73 ± 0.05^b^	2.03 ± 0.02^a^	4.09 ± 0.06^b^	19.49 ± 0.41^b^	117.92	493.38
Sea buckthorn leaves	13.25 ± 0.12^c^	3.49 ± 0.04^a^	16.38 ± 0.27^b^	3.96 ± 0.09^b^	16.86 ± 0.35^a^	164.39	687.81
Rosehip	8.36 ± 0.13^b^	5.21 ± 0.06^c^	1.51 ± 0.08^a^	0.82 ± 0.01^a^	42.48 ± 0.47^c^	172.72	722.66
P_AC	34.55 ± 0.06^c^	1.05 ± 0.03^a^	8.61 ± 0.15^a^	4.35 ± 0.18^b^	51.49 ± 3.28^a^	266.68	1114.72
P_AC + S	26.9 ± 0.03^a^	1.10 ± 0.05^ab^	8.13 ± 0.18^a^	4.00 ± 0.13^b^	59.87 ± 3.78^a^	291.16	1217.04
P_AC + SP	31.22 ± 0.05^b^	1.15 ± 0.02^b^	9.22 ± 0.23^a^	2.97 ± 0.1^a^	55.44 ± 3.55^a^	271.51	1134.91
M_A	20.00 ± 0.56^a^	0.19 ± 0.02^a^	0.80 ± 0.05^a^	0.01 ± 0.00^a^	70.10 ± 0.41^a^	283.69	1186.96
M_S + R	25.00 ± 0.27^b^	0.30 ± 0.03^ab^	0.80 ± 0.10^a^	0.01 ± 0.00^a^	70.20 ± 0.58^a^	284.09	1188.63
M_AB+S	24.00 ± 0.32^b^	0.31 ± 0.05^b^	0.80 ± 0.06^a^	0.01 ± 0.00^a^	69.60 ± 0.49^a^	281.69	1178.59
M_AC + S	25.00 ± 0.47^b^	0.29 ± 0.05^ab^	0.80 ± 0.07^a^	0.01 ± 0.00^a^	68.50 ± 0.16^a^	277.29	1160.18

P_AC, P_AC + S, and P_AC + SP refer to pastille samples prepared with apple and cranberry puree, apple and cranberry puree plus St John’s worth and sea buckthorn leaves, apple and cranberry puree plus St John’s worth, sea buckthorn leaves, and probiotic, respectively. M_A, M_S + R, M_AC + S, and M_AB + S refer to marshmallow samples prepared with apple puree, strawberry puree plus rosehip, apple and cranberry puree plus St John’s worth, and apple and blueberry puree plus sea buckthorn leaves, respectively. Different lowercase letters indicate significant differences (*p* ≤ 0.05) among the mean values of medicinal herbs (1–3 rows), among the mean values of pastille samples (4–6 rows), and among the mean values of marshmallow samples (7–10 rows).

Water activity is an important parameter for confectionery products. It provides valuable insights into their microbiological properties and determines the storage conditions, significantly affecting their chemical stability (46). Generally, bacterial growth is inhibited below water activity of 0.85, while the growth of molds and yeasts is considerably slowed at approximately 0.70, with no growth occurring below water activity of 0.60 (47). The findings presented in Table 3 reveal that the water activity (a_w_) values of pastille samples decreased with the addition of St John’s wort and probiotic. These results indicate that the presence of St John’s wort alone (P_AC + S) did not lead to any change in water activity compared to the control one (P_AC). However, the addition of probiotic in the pastille samples (P_AC + SP) can reduce the percentage of moisture. Therefore, P_AC + SP had less free water, which can retard the growth of microorganisms and enhance the final product’s shelf life. Moreover, in addition to inhibiting bacterial growth, probiotics, as natural bio-inhibitors, enhance flavor (48) and preserve the original properties of the food during storage (49, 50). In contrast, the addition of medicinal herb was found to significantly increase (*p ≤ 0.05*) the water activity of the marshmallow products. This increase is attributed to the moisture-retaining effect of herbs, which can be explained by the presence of coarse cellulose in their composition (51). Furthermore, replacing apple puree with strawberry puree resulted in an increase in the moisture content of marshmallows. This finding can be likely attributed to the dry matter content of the different fruit and berries purees. Unless slight variations occur due to different formulations, all samples remained within the acceptability limits (47), suggesting that the pastille and marshmallow products are effectively protected against undesirable microbial proliferation, contributing to their overall quality and safety.

**Table 3:** Water activity values and textural parameters of functionalized pastille and marshmallow samples.

Sample code	a_w_ (−)	Hardness (N)	Adhesiveness (N·s)	Cohesiveness (−)
P_AC	0.4364 ± 0.016^a^	0.491 ± 0.081^a^	−0.348 ± 0.057^b^	0.549 ± 0.031^a^
P_AC + S	0.4372 ± 0.018^a^	0.663 ± 0.092^a^	−0.477 ± 0.081^b^	0.497 ± 0.066^a^
P_AC + SP	0.4110 ± 0.100^a^	0.974 ± 0.166^b^	−0.561 ± 0.071^a^	0.517 ± 0.067^a^
M_A	0.7285 ± 0.005^A^	3.134 ± 0.217^B^	−0.186 ± 0.028^BC^	0.468 ± 0.043^AB^
M_S + R	0.7787 ± 0.003^C^	1.399 ± 0.081^A^	−1.022 ± 0.101^A^	0.374 ± 0.025^A^
M_AB+S	0.7488 ± 0.001^B^	6.771 ± 0.174^C^	−0.144 ± 0.090^C^	0.570 ± 0.071^B^
M_AC + S	0.7520 ± 0.004^B^	2.795 ± 0.118^B^	−0.456 ± 0.022^B^	0.386 ± 0.019^AB^

P_AC, P_AC + S, and P_AC + SP refer to pastille samples prepared with apple and cranberry puree, apple and cranberry puree plus St John’s worth and sea buckthorn leaves, apple and cranberry puree plus St John’s worth, sea buckthorn leaves, and probiotic, respectively. M_A, M_S + R, M_AC + S, and M_AB + S refer to marshmallow samples prepared with apple puree, strawberry puree plus rosehip, apple and cranberry puree plus St John’s worth, and apple and blueberry puree plus sea buckthorn leaves, respectively. Different lowercase letters indicate significant differences (*p* ≤ 0.05) among the mean values of pastille samples. Different uppercase letters indicate significant differences (*p* ≤ 0.05) among the mean values of the marshmallow samples.

Interestingly, the water activity of confectionary products is not only an important parameter in determining the overall quality and stability, but also in influencing their physicochemical properties (52). Therefore, the texture analysis of pastille and marshmallow samples was performed to determine the hardness, adhesiveness, and cohesiveness parameters (Table 3).

The hardness values of confectionery products, defined as a maximum force to compress a sample, are affected by the addition of medical herbs and/or probiotic. The significantly higher value of 0.974 ± 0.166 N was observed for P_AC + SP. This behavior, in agreement with previous results of water activity, confirmed that the decreased amount of free-water resulting in the inhibition of crystallization (13) and higher energy is required for the deformation of P_AC + SP samples. Interestingly, the same trend was observed for marshmallow samples, whose hardness tended to significantly decrease due to the increase in water activity. These results support the use of fruit and berries puree and the incorporation of medical herbs as effective strategies for enhancing the functionality of confectionary products, contributing to consumer acceptance (harder pastilles and softer marshmallows) and overall product quality. Different formulations of pastille samples have no statistically significant effect on cohesiveness values, an indicator of the strength of internal bonds that form structure of the products (53). On the other hand, a slight increase in cohesiveness value were detected for M_AB+S respect to the control sample (M_A). Nonetheless, the use of strawberry or apple and cranberry purees did not imply a significant difference (*p ≤ 0.05*) compared to the apple puree. This behavior can be advantageous for releasing compounds that are present in the chewing gum matrix.

Finally, the optical properties of confectionery products have a significant effect on their overall perception and consumption (54). The color properties of pastille and marshmallow samples containing different additives were determined by examining L*, a*, b*, C* (chromaticity), and h° (hue angle; Table 4). In general, the results showed that the color parameters were significantly affected by the different formulations (*p ≤ 0.05*). L* and b* color parameters decreased by incorporating additives for both pastille and marshmallow samples; meanwhile, a* values increased with the additives’ incorporation into confectionary products. Consequently, it was observed that a decrease in sample brightness corresponded to a dark color. The decrease in the L* values of samples containing extract may affect consumer perception negatively as it reduces the brightness and attractiveness of the product. In the present study, pastille and marshmallow samples have hue angle value closer to 0, indicating that the samples tend to red color. Furthermore, the additives have a negative effect on the chromaticity (C*) values, the brightness of a material, of pastille and marshmallow samples. On the other hand, the increasing trend of C* values in the marshmallow samples with the additives may be a result of light color of the marshmallow samples.

**Table 4:** Color parameters of functionalized pastille and marshmallow samples.

Sample code	L*	a*	b*	C*	h°
P_AC	66.90 ± 1.73^a^	14.55 ± 1.26^c^	18.99 ± 1.38^b^	23.94 ± 1.69^b^	0.92 ± 0.03^b^
P_AC + S	67.48 ± 0.97^ab^	8.38 ± 0.40^b^	18.27 ± 0.69^ab^	20.11 ± 0.74^a^	1.14 ± 0.01^a^
P_AC + SP	68.28 ± 0.96^b^	7.68 ± 0.36^a^	17.52 0.89^a^	19.13 ± 0.80^a^	1.16 ± 0.03^a^
M_A	88.58 ± 1.11^D^	0.44 ± 0.16^A^	16.71 ± 0.93^D^	16.71 ± 0.93^A^	1.54 ± 0.01^D^
M_S + R	68.80 ± 2.65^B^	20.16 ± 1.42^C^	11.50 ± 0.92^C^	23.21 ± 1.67^C^	0.52 ± 0.01^C^
M_AB+S	64.11 ± 1.49^A^	18.28 ± 0.46^B^	0.33 ± 0.16^A^	18.28 ± 0.46^B^	0.02 ± 0.01^A^
M_AC + S	74.85 ± 1.80^C^	20.68 ± 1.26^C^	8.74 ± 1.19^B^	22.46 ± 1.60^C^	0.40 ± 0.03^B^

P_AC, P_AC + S, and P_AC + SP refer to pastille samples prepared with apple and cranberry puree, apple and cranberry puree plus St John’s worth and sea buckthorn leaves, apple and cranberry puree plus St John’s worth, sea buckthorn leaves, and probiotic, respectively. M_A, M_S + R, M_AC + S, and M_AB + S refer to marshmallow samples prepared with apple puree, strawberry puree plus rosehip, apple and cranberry puree plus St John’s worth, and apple and blueberry puree plus sea buckthorn leaves, respectively. Different lowercase letters indicate significant differences (*p* ≤ 0.05) among the mean values of pastille samples. Different uppercase letters indicate significant differences (*p* ≤ 0.05) among the mean values of the marshmallow samples.

The color differences expressed as ΔE point to the relevance of color differences between samples of the same group (Table 5). The color difference between pastille control (P_AC) and the others two pastilles could have been seen even by an inexperienced observer, because ∆E exceeded 2. Meanwhile, no changes that cause color differences between functionalized pastille without and with probiotic have been distinguished by an experienced observer (∆E < 2). On the other hand, based on ∆E, the color parameters of marshmallows provided that the incorporation of additives highly affects the color difference with respect to the control. Nonetheless, conventional marshmallows are characterized by white color with no light transmission (55). Considering the growing interest in utilizing pigments for their technological advantages and quality indicators of confectionery products (18), the development of colored pastille and marshmallows is favorable. As in other confectionery products, synthetic colorants are usually used even if they pose potential risks to human health (56). Therefore, based on the research results, it is possible to improve the color characteristics of confectionery products with natural plant-based color sources, such as fruit and berries purees.

**Table 5:** Color variation (ΔE) between pastille and marshmallow samples.

	P_AC	P_AC + S		P_AC + SP
P_AC	–	6.24 ± 1.34		7.17 ± 1.28
P_AC + S	6.24 ± 1.34	–		1.31 ± 0.20
P_AC + SP	7.17 ± 1.28	1.31 ± 0.20		–
	M_A	M_AB + S	M_AC + S	M_S + R
M_A	–	34.43 ± 0.91	25.72 ± 1.33	28.40 ± 1.99
M_AB + S	34.43 ± 0.91	–	13.85 ± 1.34	12.27 ± 1.69
M_AC + S	25.72 ± 1.33	13.85 ± 1.34	–	6.67 ± 0.91
M_S + R	28.40 ± 1.99	12.27 ± 1.69	6.67 ± 0.91	–

P_AC, P_AC + S, and P_AC + SP refer to pastille samples prepared with apple and cranberry puree, apple and cranberry puree plus St John’s worth and sea buckthorn leaves, apple and cranberry puree plus St John’s worth, sea buckthorn leaves, and probiotic, respectively. M_A, M_S + R, M_AC + S, and M_AB + S refer to marshmallow samples prepared with apple puree, strawberry puree plus rosehip, apple and cranberry puree plus St John’s worth, and apple and blueberry puree plus sea buckthorn leaves, respectively.

Considering all the aforementioned physicochemical characterization, it is obvious that the type of fruit or berries purees and the additive will have various effects on the pastille and marshmallow product properties. Modifications to the conventional pastille and marshmallow can be used as an approach to reduce calorie values and positively influence consumer preferences by providing a (i) good microbial stability, (ii) an improved textural property, and (iii) an attractive visual appearance.

### 3.2 Release of bioactive compounds from marshmallow samples during *in vitro* digestion

During the *in vitro* digestion of the samples, the amount of bioactive compounds (TPC and TAC) released from the marshmallow samples and their antioxidant activity at each digestion phase was monitored.

The amount of the bioactive compounds investigated was measured to understand how they behave during digestion, and in which part of the gastrointestinal tract they are mostly released or degraded. Berries, especially members of Rosaceae family (strawberry, raspberry, blackberry), and Ericaceae family (blueberry, cranberry), belong to the best dietary sources of bioactive compounds. Due to their antioxidant properties, these bioactive compounds are of great interest to food technologists, as they offer opportunities for the use bioactive compounds as functional food ingredients (57).

**Figure 4:**
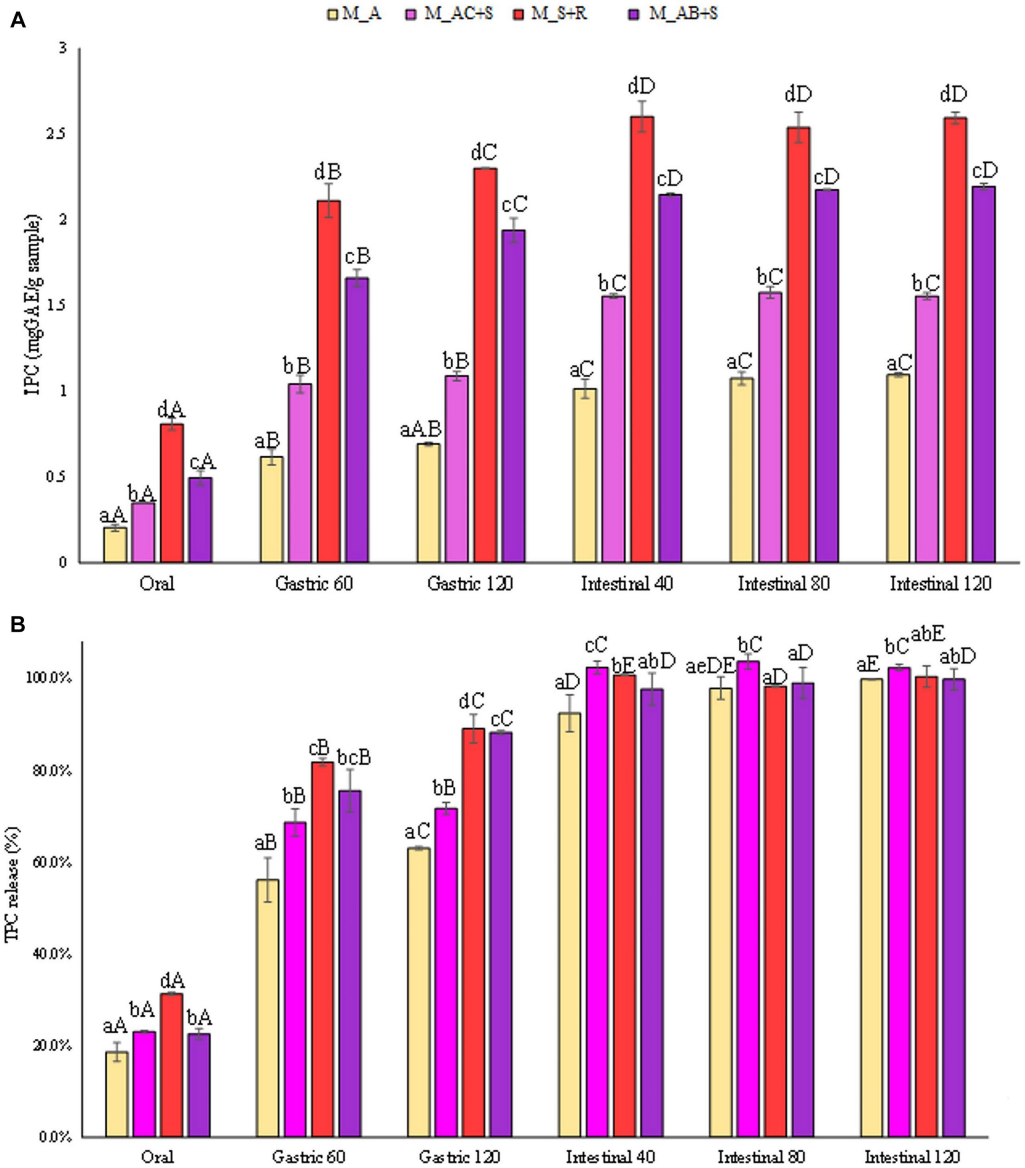
**(A)** Release of total phenolic compounds (TPC) expressed in mg_GAE_/g_sample_, and **(B)** as percentage of release during the digestive phases of the marshmallow samples. Different lowercase letters above the bars indicate significant differences (*p ≤ 0.05*) among the mean values of different samples at the same digestion phase. Different uppercase letters above the bars indicate significant differences (*p ≤ 0.05*) among the mean values of the same sample at different digestion phases. M_A, M_S + R, M_AC + S, and M_AB+S refer to marshmallow samples prepared with apple puree, strawberry puree plus rosehip, apple and cranberry puree plus St John’s worth, and apple and blueberry puree plus sea buckthorn leaves, respectively.

In Figure 4A the concentrations of TPC in terms of mg_GAE_/g_sample_ were reported as function of the advancement of digestive phases. As expected, the marshmallows prepared with the addition of apple showed the lowest level of TPC at the end of the intestinal phase (1.1 mg_GAE_/g_sample_) compared to the other samples under investigation. Specifically, the addition to the apple of cranberry and a medicinal plant such as St. John’s wort, contributed to enhancing the level of TPC in the marshmallows of about 36% (1.5 mg_GAE_/g_sample_), while the addition of blueberry and sea buckthorn leaves led to a doubled concentration of TPC in the corresponding marshmallows (2.2 mg_GAE_/g_sample_). Moreover, the marshmallows prepared with the strawberry and rosehip showed TPC level (2.6 mg_GAE_/g_sample_) comparable to that of the marshmallow containing blueberry. The highest amount of phenolic compounds released in the intestinal fluid may be partly attributed to the abundance of phenolics found in rosehip, mainly phenolic acids, which showed higher total antioxidant capacity than that of several fruits and berries including sour cherry, strawberry, raspberry (58).

The obtained results, reported in Figure 4B, showed that TPC from all the different investigated marshmallow samples are constantly released during digestion, starting from a release of 24% on average after the oral phase, passing through a release of 75% on average at the end of the gastric phase, and reaching the maximum release occurring in the intestinal phase (100%). Due to the hydrolysis of starch taking place in the intestine, the bioactives are unlocked and their release in the digestive fluid is enhanced. Previous studies (59, 60) stated that the gastric and intestinal phases cause a much higher increase in TPC indicating that the action of enzymes (pepsin, pancreatin) and pH at these stages effectively favor the release of polyphenols from the food matrix. Nonetheless, it should also be noted that the pH susceptibility of structurally different plant phenolic compounds, stored in cell vacuoles and walls, depends heavily on the phenol structure. For example, the structural characteristics of a molecule characterized by aromatic systems, due to the spatial arrangement between an –OH group and the π-electron system governing the extent of π-orbital overlap, affect the susceptibility to chemical changes, making it more resistant to pH-induced degradation.

**Figure 5:**
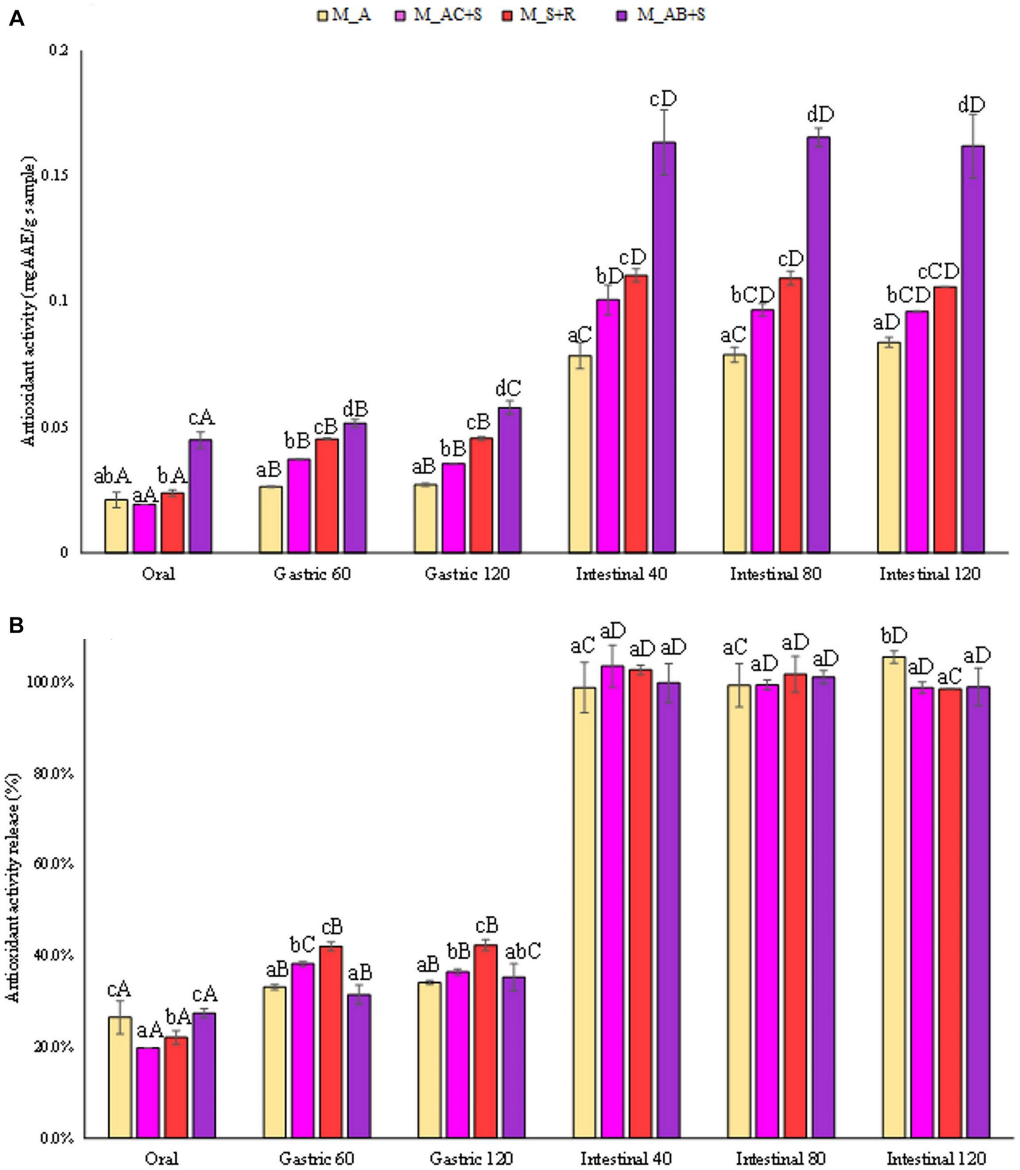
**(A)** Release of antioxidant activity (FRAP) expressed in mg_AAE_/g_sample_, and **(B)** as percentage of release during the digestive phases of the marshmallow samples. Different lowercase letters above the bars indicate significant differences (*p ≤ 0.05*) among the mean values of different samples at the same digestion phase. Different uppercase letters above the bars indicate significant differences (*p ≤ 0.05*) among the mean values of the same sample at different digestion phases. M_A, M_S + R, M_AC + S, and M_AB+S refer to marshmallow samples prepared with apple puree, strawberry puree plus rosehip, apple and cranberry puree plus St John’s worth, and apple and blueberry puree plus sea buckthorn leaves, respectively.

The results reported in Figures 5A,B showed the release in terms of antioxidant activity expressed in mg_GAAE_/g_sample_ and in percentage compared to the antioxidant activity of the completely degraded sample. The marshmallow sample functionalized with the addition of blueberry, apple, and sea buckthorn leaves showed the highest antioxidant activity, being about 60% higher than that detected in the other samples investigated at the end of the intestinal phase. Skrovankova et al. (57) stated that the total antioxidant capacity of raspberries and strawberries are similar to each other, but lower than in blueberries (57). The antioxidant activity of blueberry depends on the phytochemical complex, being mainly represented by anthocyanins, procyanidins, chlorogenic acid, and other flavonoid compounds. It is supposed that the major contributors to their antioxidant activity are mainly anthocyanins, responsible for about 84% of antioxidant activity. Nevertheless, it should also be highlighted that the antioxidant capacity of berries is influenced by several factors such as cultivar., genotype, variety, growing location, cultivation techniques, pre-harvest climate conditions, processing, and storage (57). However, the release (%) of antioxidants from all the marshmallow samples studied showed a similar trend during the gastrointestinal digestive stages (Figure 5B).

**Figure 6:**
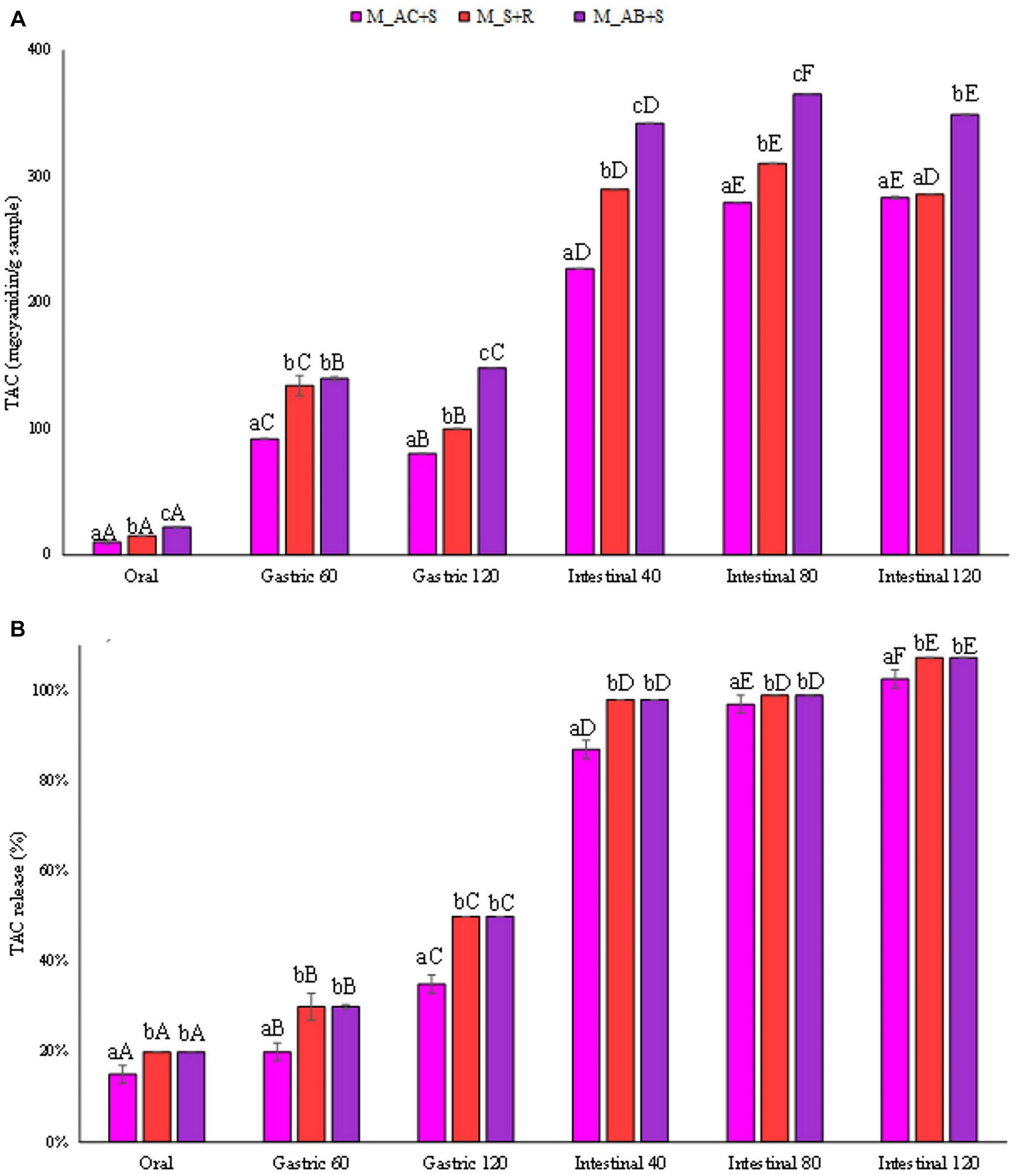
**(A)** Release of total anthocyanin content (TAC) expressed in mg_cyanidin 3-o-glucoside_/g_sample_, and **(B)** as percentage of release during the digestive phases of the marshmallow samples. Different lowercase letters above the bars indicate significant differences (*p ≤ 0.05*) among the mean values of different samples at the same digestion phase. Different uppercase letters above the bars indicate significant differences (*p ≤ 0.05*) among the mean values of the same sample at different digestion phases. M_A, M_S + R, M_AC + S, and M_AB+S refer to marshmallow samples prepared with apple puree, strawberry puree plus rosehip, apple and cranberry puree plus St John’s worth, and apple and blueberry puree plus sea buckthorn leaves, respectively.

The amount of TAC (mg_cyanidin 3-o-glucoside_/g_sample_) released from the marshmallow samples studied during the digestives phases was reported in Figure 6A. Anthocyanins in berries are the major known polyphenolic compounds, responsible for fruit color, and can be used as natural pigments (red and blue colors) for the food industry. The marshmallow prepared with the addition of blueberry, consistently with the antioxidant activity values, showed the highest TAC in the intestinal fluid at the end of the digestion than that detected for the other marshmallow samples (360, 290, 283 mg_cyanidin 3-o-glucoside_/g_sample_, for marshmallows containing blueberries, strawberries, and cranberries, respectively), reaching in all the cases the highest release (100%, Figure 6B).

Studies collectively demonstrate a high variation among blueberry cultivars for total anthocyanin content, with values ranging from 19.3 to 677.8 mg_cyanidin 3-o-glucoside_ per 100 g fresh weight (61).

Although the anthocyanin content of strawberries, compared to other common berries, such as blueberries, blackberries, and raspberries, is much lower, the addition of rosehip in the preparation of marshmallows could have contributed in the total anthocyanin content of the sample since rosehip also contains anthocyanins (57).

### 3.3 Release of bioactive compounds from pastille samples during *in vitro* digestion

**Figure 7:**
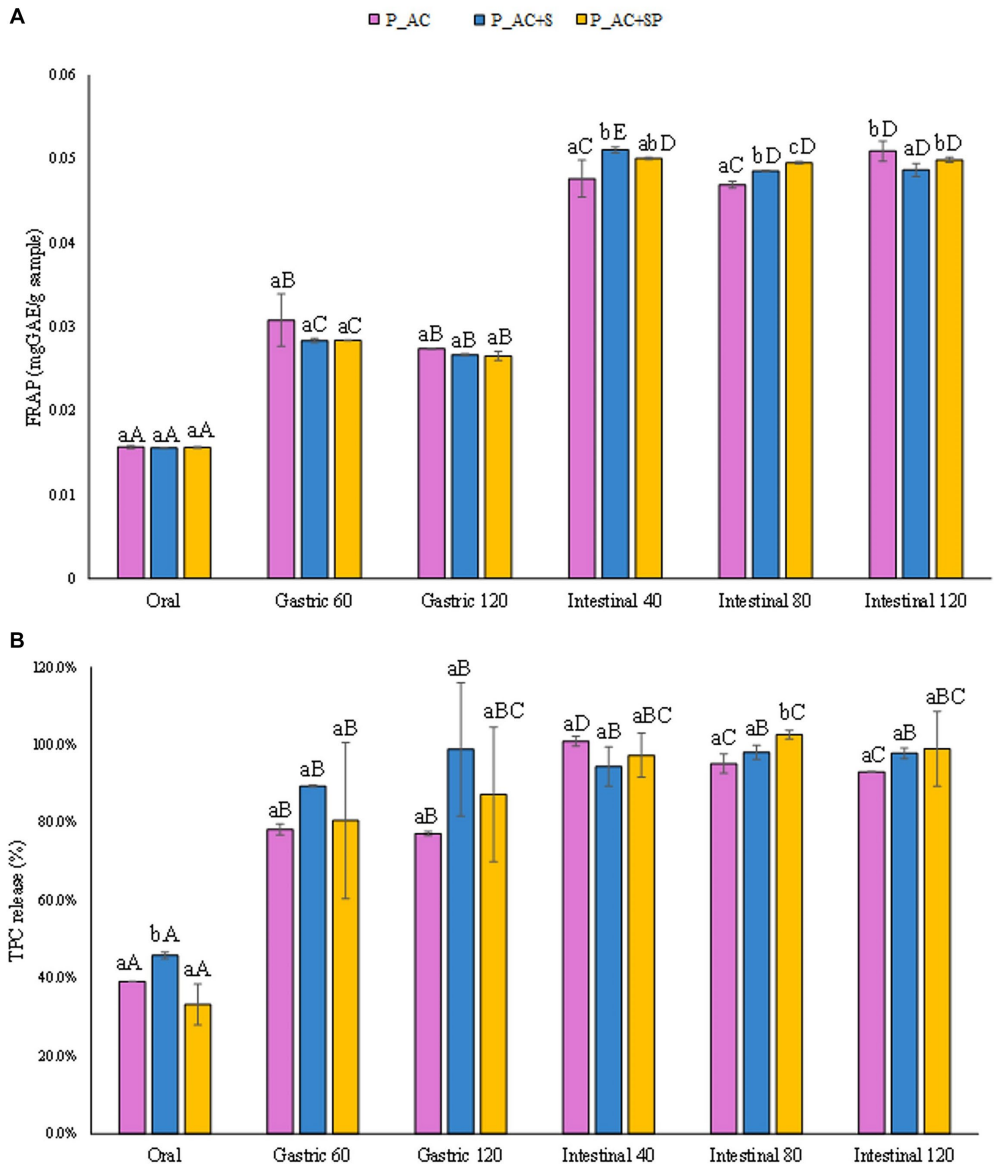
**(A)** Release of total phenolic compounds (TPC) expressed in mg_GAE_/g_sample_, and **(B)** as percentage of release during the digestive phases of the pastille samples investigated. Different lowercase letters above the bars indicate significant differences (*p ≤ 0.05*) among the mean values of different samples at the same digestion phase. Different uppercase letters above the bars indicate significant differences (*p ≤ 0.05*) among the mean values of the same sample at different digestion phases. P_AC, P_AC + S, and P_AC + SP refer to pastille samples prepared with apple and cranberry puree, apple and cranberry puree plus St John’s worth and sea buckthorn leaves, apple and cranberry puree plus St John’s worth, sea buckthorn leaves, and probiotic, respectively.

In Figure 7A the concentrations of TPC released from the pastille samples (mg_GAE_/g_sample_) as function of the advancement of digestive phases were reported. The obtained results demonstrated that the TPC detected after the gastrointestinal digestion of the pastilles prepared with apple and cranberry were slightly lower (by 30%) than that observed for the samples containing not only apple and cranberry purees but also sea buckthorn, St. John’s wort and probiotic. Moreover, results reported in Figure 7B, showed that TPC from all the different investigated samples are released during digestion, starting from a release of 40% on average after the oral phase, passing through a release of 87% on average at the end of the gastric phase, and reaching the maximum release after the intestinal phase (97%). These findings, which highlighted the faster and less controlled release of TPC from pastille samples during digestion compared to that observed for marshmallow samples, could be attributed to the less compact structure of the pastille samples with respect to the dense and gummy marshmallows’ structure.

**Figure 8:**
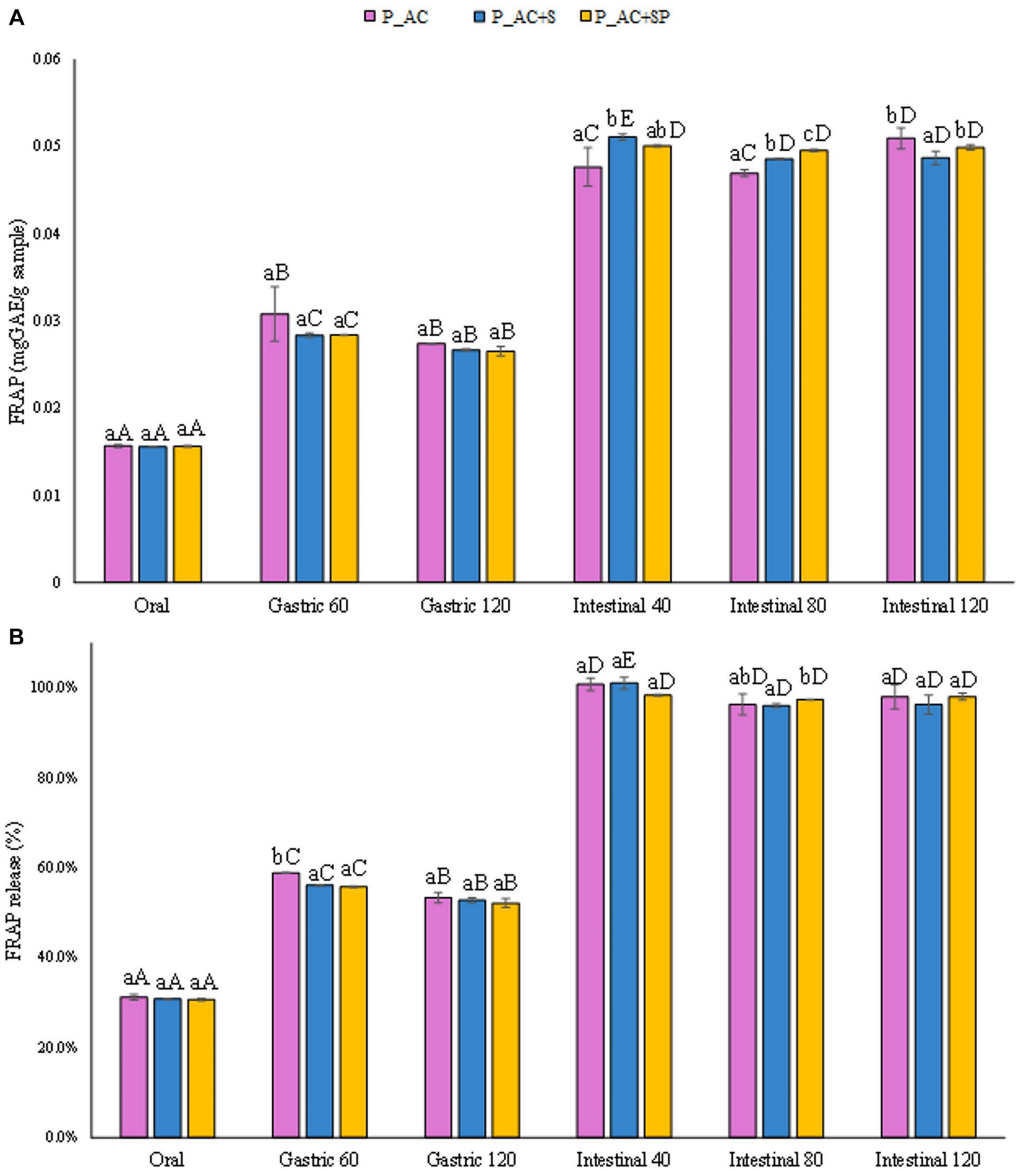
**(A)** Release of antioxidant activity (FRAP) expressed in mg_AAE_/g_sample_, and **(B)** as percentage of release during the digestive phases of the pastille samples investigated. Different lowercase letters above the bars indicate significant differences (*p ≤ 0.05*) among the mean values of different samples at the same digestion phase. Different uppercase letters above the bars indicate significant differences (*p ≤ 0.05*) among the mean values of the same sample at different digestion phases. P_AC, P_AC + S, and P_AC + SP refer to pastille samples prepared with apple and cranberry puree, apple and cranberry puree plus St John’s worth and sea buckthorn leaves, apple and cranberry puree plus St John’s worth, sea buckthorn leaves, and probiotic, respectively.

A similar behavior was observed for the release in terms of antioxidant activity, both expressed in mg_AAE_/g_sample_ (Figure 8A) and as percentage of antioxidant activity released (Figure 8B), from the pastille samples during the digestion phases. The results showed that no significant differences (*p ≤ 0.05*) in terms of antioxidant activity were detected among pastilles prepared with the addition of apple, cranberry, sea buckthorn, and St. John’s wort, pastilles prepared with the addition of apple, cranberry, sea buckthorn, St. John’s wort, and probiotic, and the control sample prepared with the addition of apple and cranberry.

**Figure 9:**
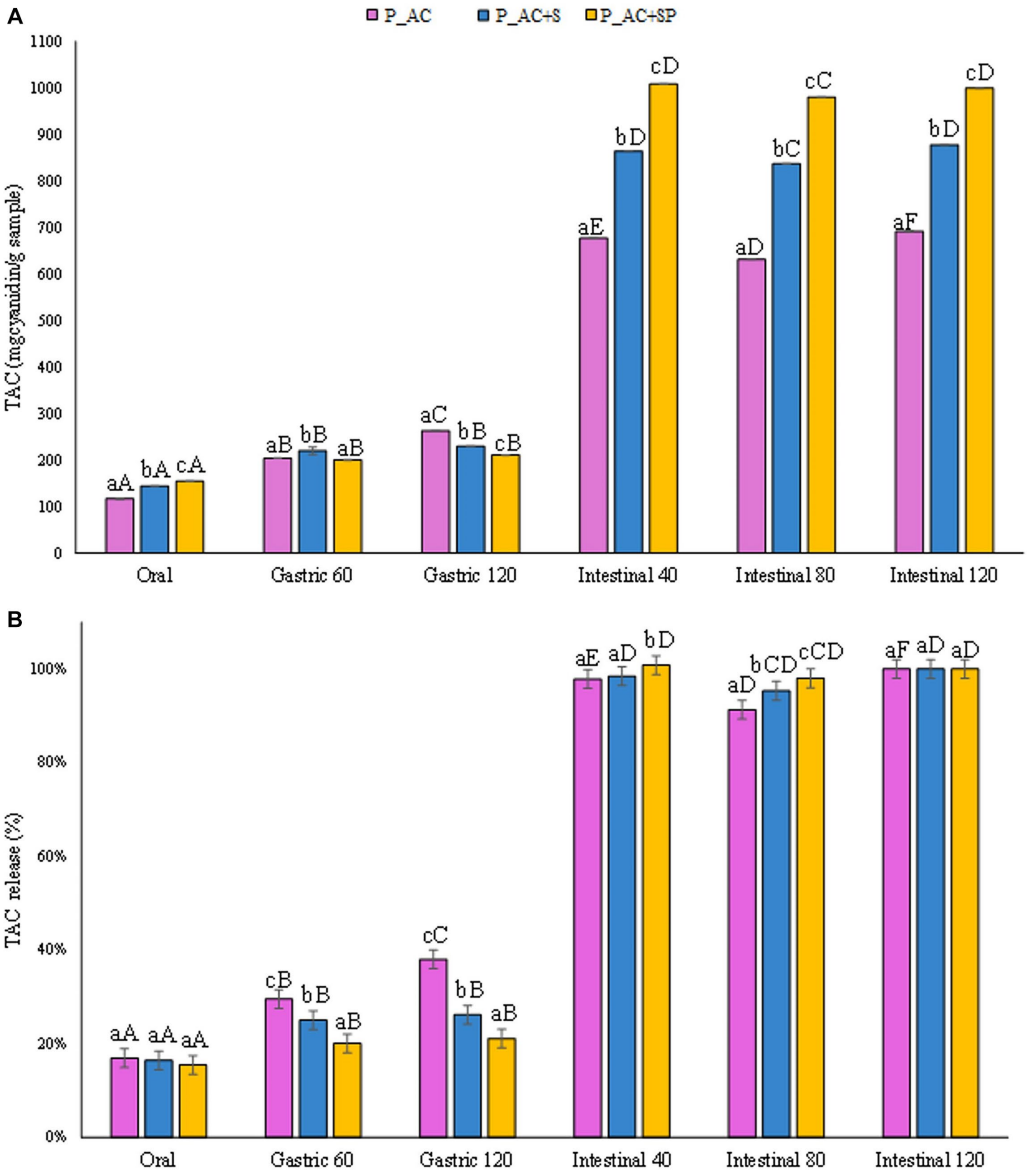
**(A)** Release of total anthocyanin content (TAC) expressed in mg_cyanidin 3-o-glucoside_/g_sample_, and **(B)** as percentage of release during the digestive phases of the pastille samples investigated. Different lowercase letters above the bars indicate significant differences (*p ≤ 0.05*) among the mean values of different samples at the same digestion phase. Different uppercase letters above the bars indicate significant differences (*p ≤ 0.05*) among the mean values of the same sample at different digestion phases. P_AC, P_AC + S, and P_AC + SP refer to pastille samples prepared with apple and cranberry puree, apple and cranberry puree plus St John’s worth and sea buckthorn leaves, apple and cranberry puree plus St John’s worth, sea buckthorn leaves, and probiotic, respectively.

Interestingly, results reported in Figure 9A demonstrated that the pastilles prepared with the addition of sea buckthorn, St. John’s wort, and probiotic had the highest total anthocyanin content released after the gastrointestinal digestion (1,000 mg_cyanidin 3-o-glucoside_/g_sample_), slightly higher than the TAC level detected for the pastilles prepared without the addition of probiotic (14%), and the control sample (44%). The results showed that the probiotic, for its protective activity on the intestinal bacterial flora, could increase the release of anthocyanins during digestion, improving their stability and bioaccessibility, and showing a good potential to modulate the gut bacterial flora during the digestive processes (62). Indeed, as reported in Figure 9B, the addition of the probiotic to the formulation led to a more controlled release of TAC from the sample during the oral and gastric phases, leading to a better release during the intestinal phases compared to the other investigated samples.

### 3.4 Sensory evaluation

**Figure 10:**
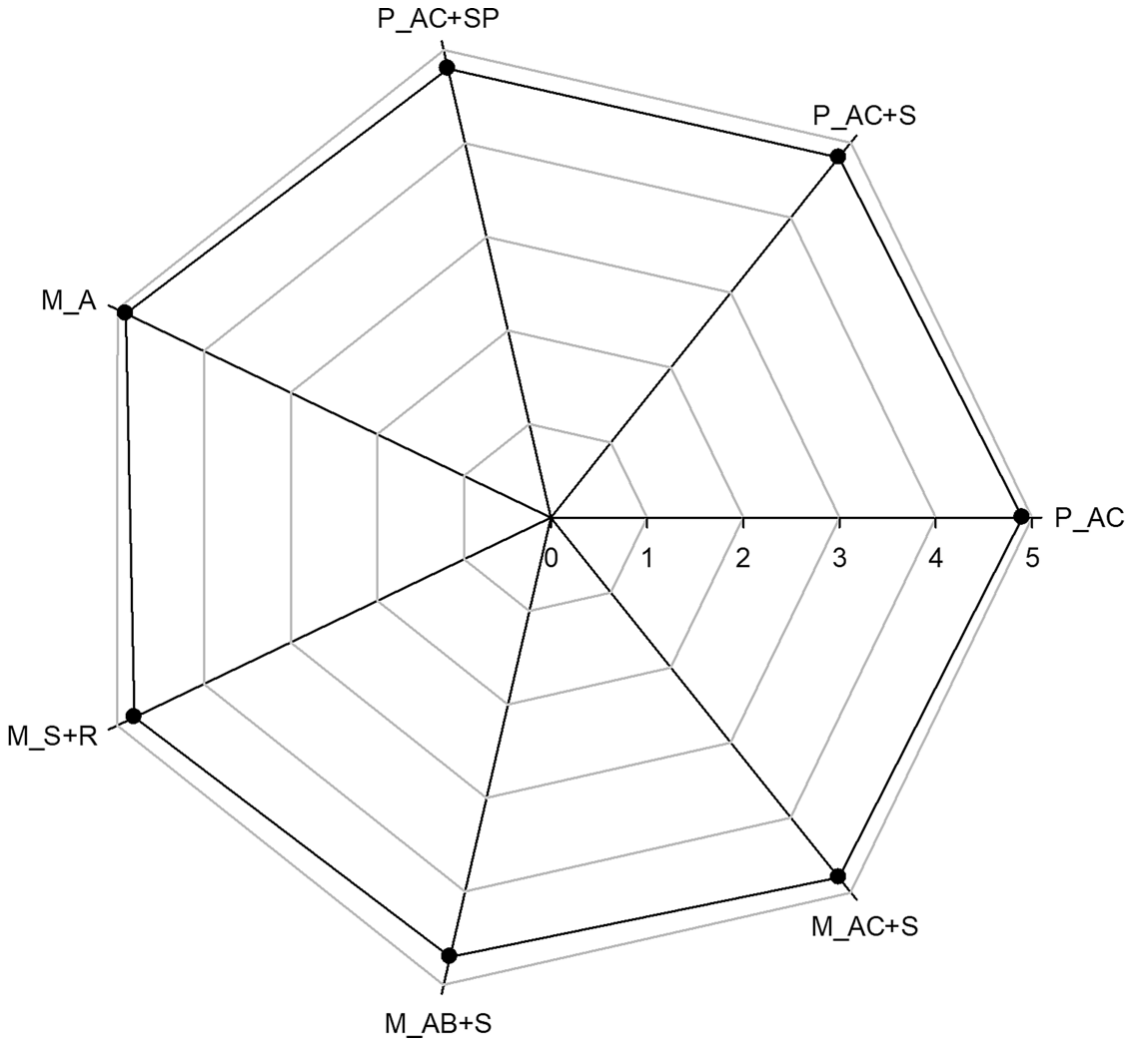
Sensory final evaluation for the pastille and marshmallow samples. P_AC, P_AC + S, and P_AC + SP refer to pastille samples prepared with apple and cranberry puree, apple and cranberry puree plus St John’s worth and sea buckthorn leaves, apple and cranberry puree plus St John’s worth, sea buckthorn leaves, and probiotic, respectively. M_A, M_S + R, M_AC + S, and M_AB+S refer to marshmallow samples prepared with apple puree, strawberry puree plus rosehip, apple and cranberry puree plus St John’s worth, and apple and blueberry puree plus sea buckthorn leaves, respectively.

Sensory features are one of the determinants of a consumer’s choice of food (63). Sensory evaluation of confectionery products was conducted using the 5-point scale. The highest values (4.9 pts) were scored by the pastille and marshmallow without the addition of functional ingredients (control samples; Figure 10) due to the vaguely grassy sensation present in the other products. Pastille samples resulted homogeneously dried without any defects on the surface, soft with uniform and finely porous structure and without coarse hardening on the side edges. Moreover, the appearance of P_AC + SP is comparable to the that of P_AC + S, as confirmed by color analysis in section 3.1. As a result, the addition of probiotic to pastille formulation did not cause any changes in the final evaluation score. Instead, the lowest score (4.7 pts) was achieved by M_AB+S due to the greater intensity and darker color of blueberry puree compared to the strawberry and cranberry ones. Overall, marshmallow samples were characterized by firm, foamy, uniform structure without defects, and rounded shape characteristic of these products without rough hardening.

## 4 Conclusion

The results showed that the physicochemical properties of functionalized pastille and marshmallow samples are consistent with traditional confectionery products, with acceptable water activity (< 0.85) levels for microbiological safety. The release of bioactive compounds (total phenolic compounds and total anthocyanin) during *in vitro* digestion was a crucial aspect of this study. Results showed that marshmallows containing bioactive compounds exhibited increased total phenolic and anthocyanin content, especially when blueberry puree and rosehip were added, with the highest release in the intestinal phase. Similarly, antioxidant activity increased in functionalized marshmallows respect to the control one and the release of antioxidants during digestion was consistent across all marshmallow samples, indicating that the digestive process effectively released polyphenols from the food matrix. In pastille samples, total phenols content was released during the *in vitro* digestion, with the highest release in the intestinal phase. Interestingly, the addition of probiotics in pastille formulations led to a more controlled release of anthocyanin during digestion, potentially improving stability and bioaccessibility.

In summary, this research demonstrates the feasibility of developing gluten-free confectionery products enriched with natural functional ingredients that offer potential health benefits. Moreover, the release of bioactive compounds during digestion underscores their potential as immune-boosting and naturally composed food products. Nonetheless, further studies are required to exploit the microbial stability, organoleptic properties, and sensory analysis of the new developed functional confectionery products.

## Data availability statement

The original contributions presented in the study are included in the article/supplementary material, further inquiries can be directed to the corresponding authors.

## Ethics statement

Ethical approval was not required for the study involving humans in accordance with the local legislation and institutional requirements. Written informed consent to participate in this study was not required from the participants or the participants’ legal guardians/next of kin in accordance with the national legislation and the institutional requirements.

## Author contributions

YP: Funding acquisition, Investigation, Writing – original draft, Writing – review & editing. OB: Conceptualization, Visualization, Writing – review & editing. ZN: Data curation, Funding acquisition, Investigation, Supervision, Writing – review & editing. AP: Conceptualization, Data curation, Formal analysis, Investigation, Methodology, Visualization, Writing – original draft, Writing – review & editing. SC: Conceptualization, Data curation, Formal analysis, Investigation, Methodology, Visualization, Writing – original draft, Writing – review & editing. GF: Conceptualization, Resources, Supervision, Writing – review & editing. EB: Conceptualization, Methodology, Writing – review & editing. AB: Conceptualization, Visualization, Writing – review & editing.
